# D-2-Hydroxyglutaric Aciduria with Enchondromatosis and Angiokeratoma Circumscriptum

**DOI:** 10.7759/cureus.6157

**Published:** 2019-11-14

**Authors:** Allie Preston, Kara Reardon, Neil Crowson, Walter Lamar, Jason M Hirshburg

**Affiliations:** 1 Internal Medicine, Baylor University Medical Center, Dallas, USA; 2 Dermatology, Oklahoma University Health Sciences Center, Oklahoma City, USA; 3 Dermatopathology, Regional Medical Laboratory, Tulsa, USA

**Keywords:** enchondromatosis, d-2-hydroxyglutaric aciduria, angiokeratoma, somatic mosaicism

## Abstract

In this study, we report a four-year-old male with D-2-hydroxyglutaric aciduria (D2HA) and enchondromatosis with a prior history of hyperpigmented, segmental whorls and streaks on his abdomen who later presented with an eruption of angiokeratoma circumscriptum within a similar distribution. His condition can likely be explained by underlying somatic mosaicism; however, a unifying culprit gene mutation has not yet been identified. To date, only 10 reported cases of D2HA with enchondromatosis are available in the literature with three reported skin findings. This is the first reported case of angiokeratoma circumscriptum associated with the rare condition of D2HA and enchondromatosis.

## Introduction

D-2-hydroxyglutaric aciduria (D2HA) with enchondromatosis is a neurometabolic and skeletal disorder that has rarely been described in the literature in sporadic patients [1]. It is characterized by developmental delay, epilepsy, hypotonia, and dysmorphic features, in addition to multiple benign masses of hyaline cartilage in the medulla of metaphyseal bone. Heterozygous variants in isocitrate dehydrogenase 1-gene or isocitrate dehydrogenase 2-gene are associated with this disorder, with molecularly characterized cases appearing to be mosaic [1].

## Case presentation

**Figure 1:**
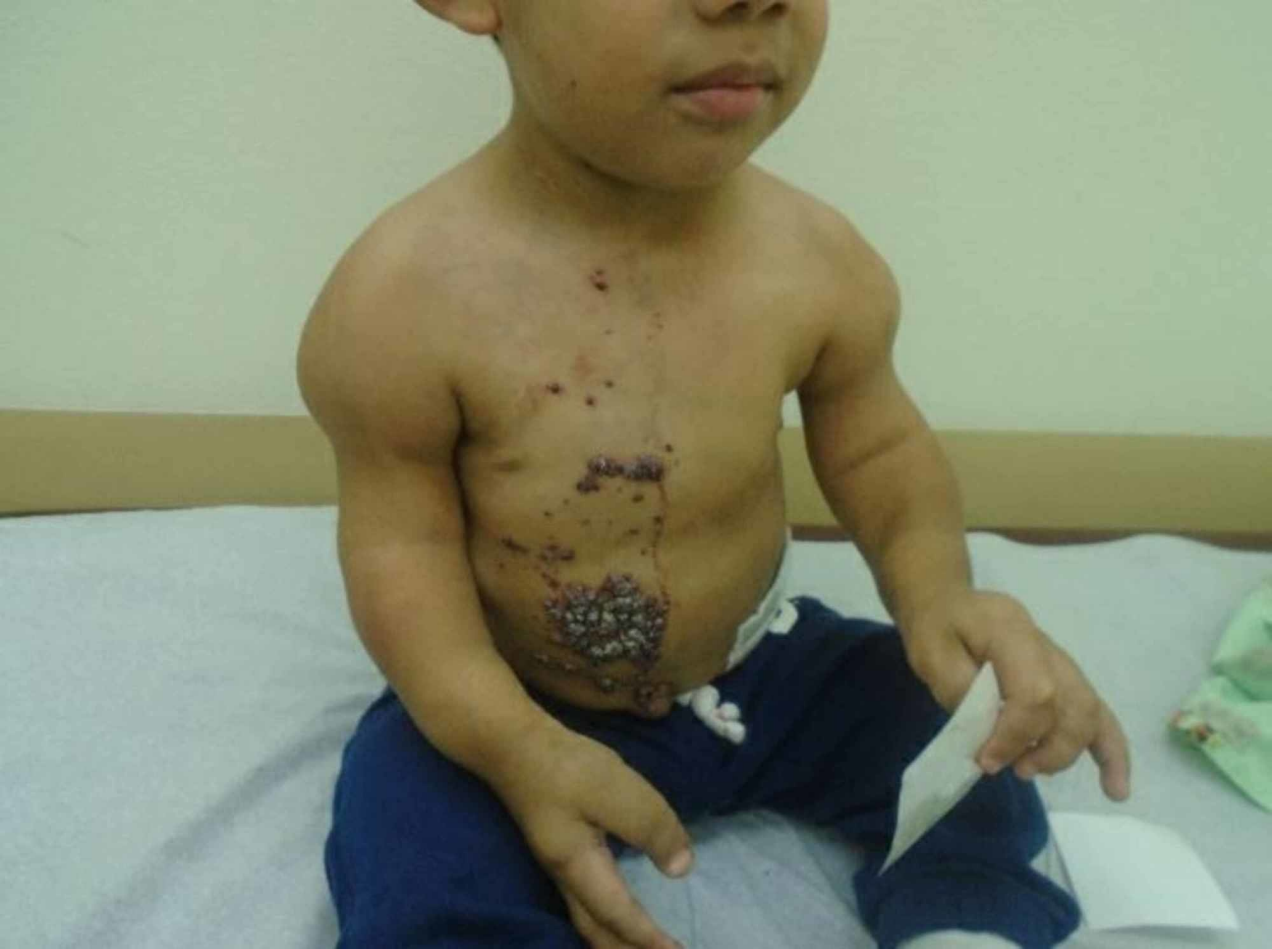
Hyperkeratotic, dark red vascular appearing papules coalescing into plaques on chest and abdomen

A four-year-old male with D2HA with enchondromatosis and acute myelogenous leukemia (AML) in remission presented to our clinic with a one-year history of hyperkeratotic, dark red papules coalescing into plaques primarily on his lower chest and upper abdomen (Figure 1). These lesions were asymptomatic except for occasional bleeding after trauma.

**Figure 2:**
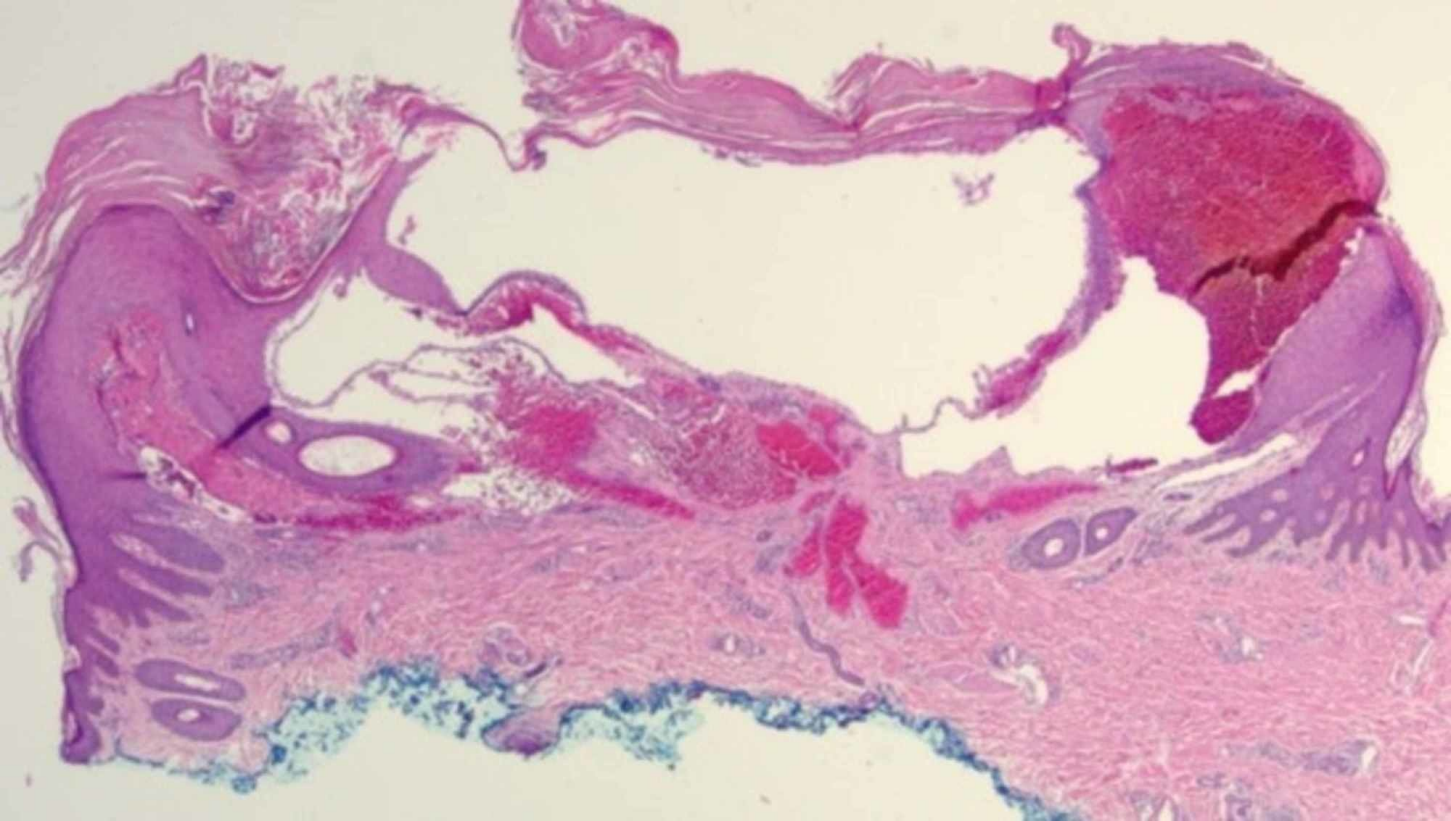
Hyperkeratosis with dilated, blood-filled, endothelial-lined spaces present immediately beneath the epidermis

Biopsy of one of the lesions demonstrated marked hyperkeratosis with dilated, blood-filled, endothelial-lined spaces presenting immediately beneath the epidermis, consistent with an angiokeratoma (Figure 2). Due to the asymptomatic nature of the angiokeratomas, treatment was not performed.

This case was presented as an oral presentation at the American Academy of Dermatology 2019 Annual Meeting (Allie Preston, Kara Reardon, Neil Crowson, Walter Lamar, Jason M. Hirshburg. D-2-Hydroxyglutaric Aciduria with Enchondromatosis and Angiokeratoma Circumscriptum. J Am Acad Dermatol. 2019, 81:AB41. https://www.jaad.org/article/S0190-9622(19)31186-7/fulltext).

## Discussion

D2HA with enchondromatosis is a rare condition that, to our knowledge, has only been reported in 10 other patients [1]. It has been suggested that somatic mutations in IDH1 may explain the association between enchondromatosis and D2HA [2-3]. Mutations in IDH1 (isocitrate dehydrogenase) result in cellular depletion of α-ketoglutarate and increased production of D-2-hydroxyglutarate (leading to spillage of D-2-hydroxyglutaric acid in the urine), resulting in over-activation of the HIF-1a pathway that can lead to uncontrolled proliferation of chondrocytes [2]. Despite having a positive IDH1 mutation in his AML, genetic testing failed to reveal an IDH1 mutation in our patient’s peripheral blood, and further testing of the biopsied angiokeratoma was also negative for an IDH1 mutation. Only three of the 10 cases in the literature report dermatologic manifestations associated with D2HA with enchondromatosis: two with hemangiomas and another with hyperpigmented skin discoloration with whorls and streaks along lines of Blaschko [1,4]. None reported angiokeratomas as an associated finding.

**Figure 3:**
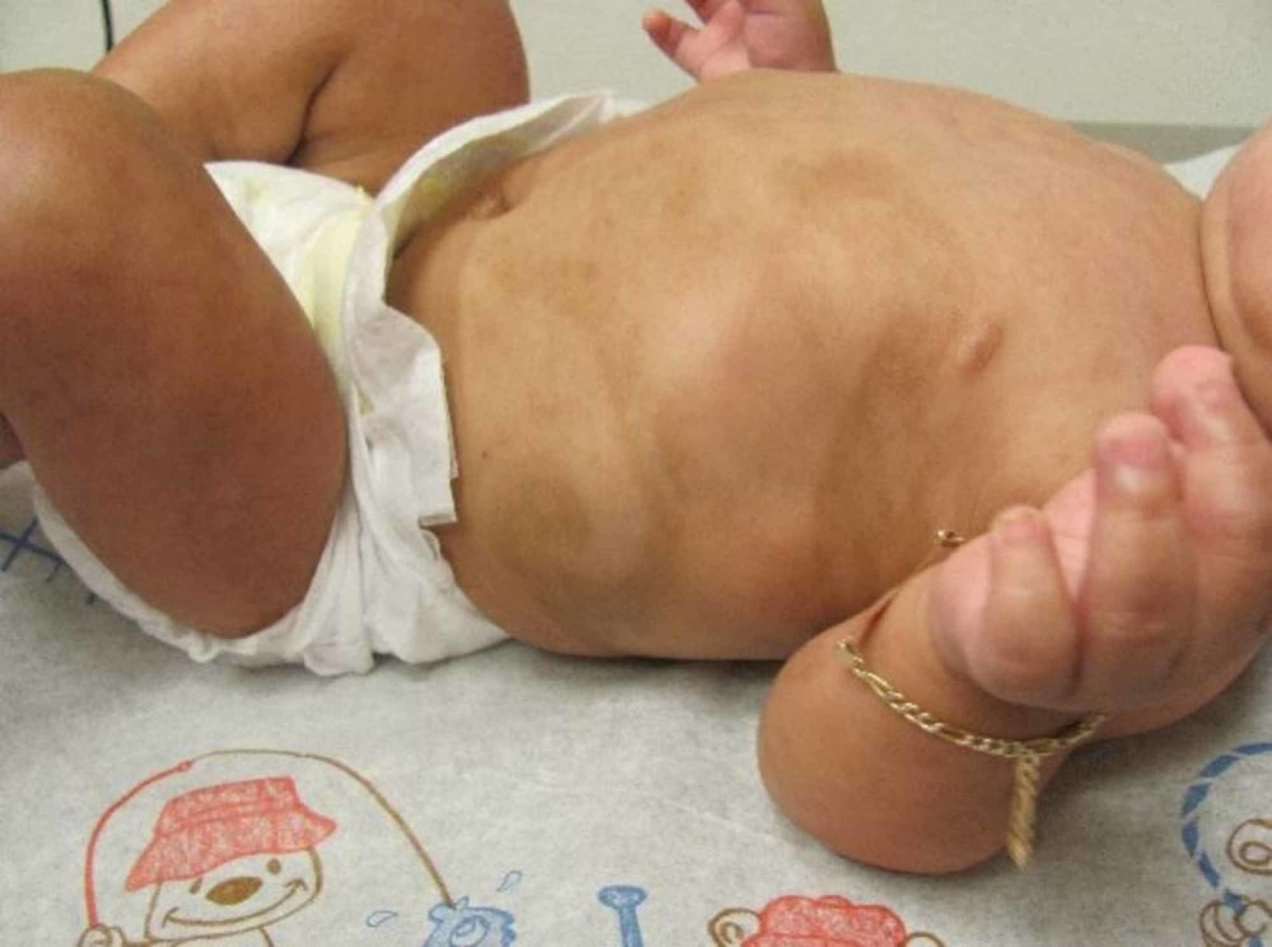
Segmental, streaked, and whorled, hyperpigmented patches

Interestingly, our patient had previously presented to our dermatology clinic at seven months of age with segmental, streaked and whorled, hyperpigmentation over his trunk that was suspected to be an expression of genetic mosaicism (Figure 3).

At that time, he was referred to genetics for further evaluation and biopsies were not performed (in accordance with his parent’s wishes). However, due to the similar distribution, it is unclear whether the angiokeratomas possibly evolved from these early lesions or arose independently as the patient was lost to dermatological follow-up until he presented to our clinic again at four years of age.

Angiokeratoma circumscriptum neviforme is the rarest type of angiokeratoma, initially described by Fabry in 1915, and typically presents more commonly in females (3:1) in a linear or segmental distribution most commonly on the lower extremities and gluteal region, but it has also been reported on the neck, arm, and trunk [5-6]. As in our patient, the angiokeratoma circumscriptum lesions are typically localized to small areas (though they have been reported to cover up to one-quarter of the body) and can bleed with minor trauma but are otherwise asymptomatic [6]. Likewise, in keeping with the proposition by Bechara et al., the band-like distribution of lesions, as well as the history of segmental hyperpigmented whorls and streaks as an infant in our case, strongly supports the diagnosis of underlying somatic mosaicism [7].

Though the exact etiology of our patient’s condition has not yet been elucidated, it is important to document the association of his D2HA and enchrondromatosis with the novel cutaneous finding of angiokeratoma circumscriptum. Additionally, this is the second patient in the literature with D2HA and enchondromatosis with the cutaneous finding of whorled pigmentation. This seems to indicate that linear, whorled hyperpigmenation may be an associated finding of D2HA and enchondromatosis. Our patient’s family opted not to cosmetically treat his dermatologic manifestations; however, multiple options for treating angiokeratoma circumscriptum neviforme include cryotherapy, electrocoagulation, curettage, lasers, and surgery [5].

## Conclusions

In our opinion, his current lesions most likely represent angiokeratoma circumscriptum due to their well-demarcated, plaque-like appearance. This case is of particular interest because it represents a novel cutaneous finding of angiokeratoma circumscriptum associated with D2HA and enchondromatosis.
